# A Strategy for Rapid Discovery of Marker Peptides Associated with Fibrinolytic Efficacy of *Pheretima aspergillum* Based on Bioinformatics Combined with Parallel Reaction Monitoring

**DOI:** 10.3390/molecules27092651

**Published:** 2022-04-20

**Authors:** Ting-Ting Feng, Jing-Xian Zhang, Yong-Peng Zhang, Jian Sun, Hong Yu, Xiang Tao, Xiu-Hong Mao, Qing Hu, Shen Ji

**Affiliations:** 1 NMPA Key Laboratory for Quality Control of Traditional Chinese Medicine, Shanghai Institute for Food and Drug Control, 1500 Zhangheng Road, Shanghai 201203, China; ddf1227@163.com (T.-T.F.); zhjx_2003@163.com (J.-X.Z.); sunjian_0000@163.com (J.S.); 18801900083@163.com (H.Y.); tx19970427@126.com (X.T.); maoxh71@163.com (X.-H.M.); 2 China State Institute of Pharmaceutical Industry, 285 Gebaini Road, Shanghai 201203, China; 3 Institute of Traditional Chinese Medicine, Hebei North University, 11 Diamond South Road, Zhangjiakou 075000, China; zyp6081@126.com

**Keywords:** *Pheretima aspergillum*, lumbrokinase, parallel reaction monitoring, bioinformatics, fibrinolytic efficacy

## Abstract

Quality control of animal-derived traditional Chinese medicines has improved dramatically as proteomics research advanced in the past few decades. However, it remains challenging to identify quality attributes with routine proteomics approaches since protein with fibrinolytic activity is rarely reported in pheretima, a typical animal-derived traditional medicine. A novel strategy based on bioinformatics combined with parallel reaction monitoring (PRM) was developed here to rapidly discover the marker peptides associated with a fibrinolytic effect. Potential marker peptides were found by lumbrokinase sequences’ alignment and in silico digestion. The fibrinogen zymography was used to visually identify fibrinolytic proteins in pheretima. As a result, it was found that the fibrinolytic activity varied among different portions of pheretima. Fibrinolytic proteins were distributed regionally in the anterior and anterior-mid portion and there was no significant fibrinogenolytic activity observed in the mid-posterior and posterior portion. Finally, PRM experiments were deployed to validate and quantify selected marker peptides and a total of 11 peptides were identified as marker peptides, which could be potentially used in quality control of pheretima. This strategy provides a robust workflow to benefit the quality control of other animal-derived traditional medicines.

## 1. Introduction

Animal-derived medicines have proven to be an indispensable part of traditional Chinese medicines (TCMs) in clinical application over the past thousands of years. In the past, only endogenous small molecules were used in quality control. For many commonly used medicines, such as cordyceps, pheretima, and hirudo, old quality control methods had poor specificities and were hard to detect due to their high polarities [1,2]. Recently, proteins and peptides in TCMs studies have received more attention because of the ongoing technical development of proteomics research. Yang et al. selected 14 potential peptide markers based on enzymatic digestion followed by nano-flow liquid chromatography in tandem with orbitrap mass spectrometer detection to differentiate Asini Corii Colla, Cervi Cornus Colla, and Testudinis Carapacis ET Plastri Colla [3]. Cheng et al. screened peptide markers to distinguish five gelatins from different species [4]. It is obvious that peptides had better potential to be quality markers in quality control for animal-derived traditional medicines compared to small molecules.

Pheretima, a typical animal-derived medicine, has been recorded as a crude drugprescribed for anticoagulation, antipyretic and diuretic purposes in a form of dried powder for thousands of years in China [5,6]. There are four species specified in the Chinese Pharmacopeia (2020 edition), including *Pheretima aspergillum* (*P. aspergillum*, PA), *P.vulgaris, P. guillelmi,* and *P. pectinifera*. Peptide makers were identified based on a statistical analysis of peptides in different species of pheretima [7,8]. However, these markers might not reflect the fibrinolytic capability of pheretima. Liang reported that pheretima samples collected from TCM markets showed a significant difference in fibrinolytic activities, and some did not show activity at all [9]. Additionally, the different portions of earthworm varied in fibrinolytic activities in the agarose fibrinogen plate [10]. Therefore, efficacy-based quality assessment is urgently needed to ensure the effectiveness in the clinical applications. Although there have been a few methods available for analyzing fibrinolytic activities, specificity, sensitivity, and stability of these methods were unsatisfactory [11,12]. Using efficacy-related peptides for the quality assessment of pheretima is believed to be a better choice.

Lumbrokinases (LKs), a group of serine proteases with an amino acid sequence similar to earthworms, have been proven to have excellent potential in the clinical treatment of cardiovascular and cerebrovascular diseases, such as stroke, vascular dementia, myocardial infarction, and deep vein thrombosis [13,14,15,16]. However, LKs in pheretima have not been thoroughly researched due to the complexity of macromolecules [17]. Since LKs in different pheretima species contain highly homologous regions, it is reasonable to use the common sequences to represent fibrinolytic activities in LKs. Thereby, in this study, we proposed a strategy of homologous peptides rapid discovery using bioinformatics tools combined with parallel reaction monitoring (PRM).

PRM is a targeted proteomic methodology that takes advantage of the unique capabilities of quadrupole-Orbitrap mass spectrometers. In this approach, target precursor ions are selected in the quadrupole and fragmented in the high-energy collisional dissociation (HCD) cell, and subsequently all generated product ions are detected in parallel by a high resolution mass spectrometer [18]. The acquisition of full MS/MS spectra provided confident identification of those monitored analytes. The PRM approach offers increasing specificity and sensitivity comparable to the multiple/selective reaction monitoring method [19,20,21], and it has been primarily used in proteomics experiments for the quantitation of proteins and peptides [22,23] and biomarkers discovery [24,25]. A high-resolution accurate mass platform showed reliable performance in biological and clinical research.

**Figure 1:**
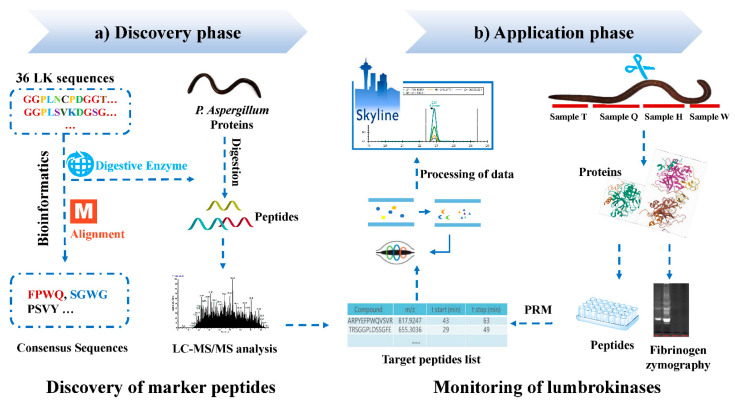
Scheme for rapid discovery of LKs and marker peptides from *P. aspergillum*. (**a**) Discovery phase: discovery of potential marker peptides based on bioinformatics tools. (**b**) Application phase: validation and evaluation of potential marker peptides representing pheretima fibrinolytic activity by PRM combined as well as fibrinogen zymography.

Label-free proteomics was widely used to both qualify and quantify peptide biomarkers. This method also has been used to identify quality markers in animal-derived traditional medicines. Previously we reported a species–specific authentication method of pheretima [8]. Unfortunately, this strategy was complicated and time-consuming, especially with multiple samples. It also requires professional knowledge of a complicated technical platform and statistical analysis. Species-specific peptides could also be predicted using bioinformatic tools via in silico digestion [26]. Thus, an integrated strategy to rapidly identify quality markers in pheretima associated with fibrinolysis was developed in this study. It consisted of two consecutive phases: firstly, the bioinformatics tools were used in LKs sequences’ alignment and in silico protein digestion to find potential fibrinolytic peptides; secondly, these peptides were validated using PRM assays. Prior to PRM, different portions of pheretima were also evaluated by fibrinogen zymography to confirm efficacy. The general workflow was shown in Figure 1. We anticipate that the strategy of quality marker peptides discovery could be implicated in other studies of unknown homologous proteins within closely related species. PRM assays for the marker peptides identification and quantitation could potentially be used in quality control of animal-derived TCMs.

## 2. Results

This study integrated two consecutive phases: discovery phase and application phase. In the discovery phase (Discovery of marker peptides), an integrated shotgun proteomics was developed, as presented in Figure 1. The bioinformatics tools were utilized for the LKs sequences’ alignment and protein digestion. LKs sequences were aligned, and conserved regions across 36 LKs sequences were pooled as the target amino acid sequences. Then, the shotgun LC-MS/MS experimental data of digested samples were validated by searching the peptide database against the PA proteins. The identified peptides containing target amino acid sequences were considered the potential markers.

In the application phase (Monitoring of LKs), fibrinolytic zymography was used to confirm fibrinolytic activities of PA. Monitoring of potential marker peptides from the discovery phase by PRM allowed rapid identification and detection of LKs from various samples.

### 2.1. Discovery of Biomarkers with Bioinformatics

#### 2.1.1. Bioinformatics

**Figure 2:**
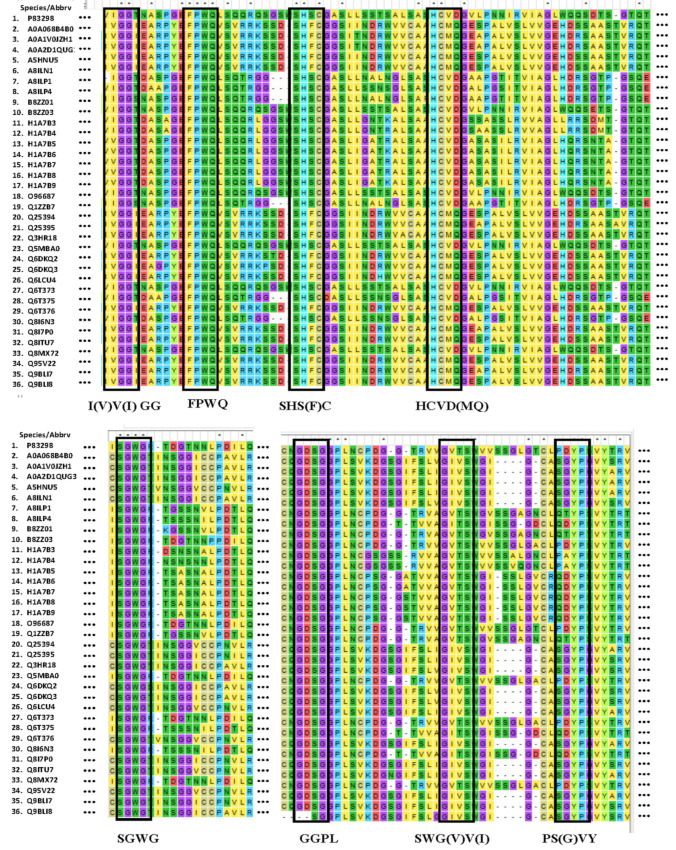
The sequence alignments of LKs. (The meaning of “⋅⋅⋅” were the amino acid residues in the LKs sequences that were not shown in the figure.) We downloaded 36 LKs sequences from different earthworm species from the UniProtKB (http://www.uniprot.org/, accessed on 10 May 2020) and U.S. National Center for Biotechnology Information (NCBI) website (https://www.ncbi.nlm.nih.gov/, accessed on 10 May 2020) and compared using the MEGA 7 software. Eight amino acid residues located were identical or similar among the different LK: I(V)V(I)GG, FPWQ, SHS(F)C, HCVD(MQ), SGWG, GGPL, SWV(G)V(I), and PS(G)VY.

LKs sequences were downloaded from the UniProtKB (http://www.uniprot.org/, accessed on 10 May 2020) and U.S. National Center for Biotechnology Information (NCBI) website (https://www.ncbi.nlm.nih.gov/, accessed on 10 May 2020) to constitute the database. The 36 LKs sequences from different earthworm species were compared using the MEGA 7 software (Figure 2). The identical amino acid residues among different LKs were marked in the same color for easy visualization. As a result, eight identical amino acid sequences were located: I(V)V(I)GG, FPWQ, SHS(F)C, HCMQ(VD), SGWG, GGPL, SWV(G)V(I), and PS(G)VY.

Trypsin is the most-used protease in shotgun proteomics research. It has been the core technology in the analysis of protein and protein–protein interactions and protein post-translational modifications [27,28]. However, LKs sequences were proline-rich sequences and had a low content of lysine and arginine. It obvious to us that trypsin was not a good choice for LKs digestion. In silico protein digestion revealed that Glu-C is a better choice for our purpose, since it specifically cleaved after acidic amino acid residues (Glu/Asp).

#### 2.1.2. Exploration on the Distribution of Fibrinolytic Activity

**Figure 3:**
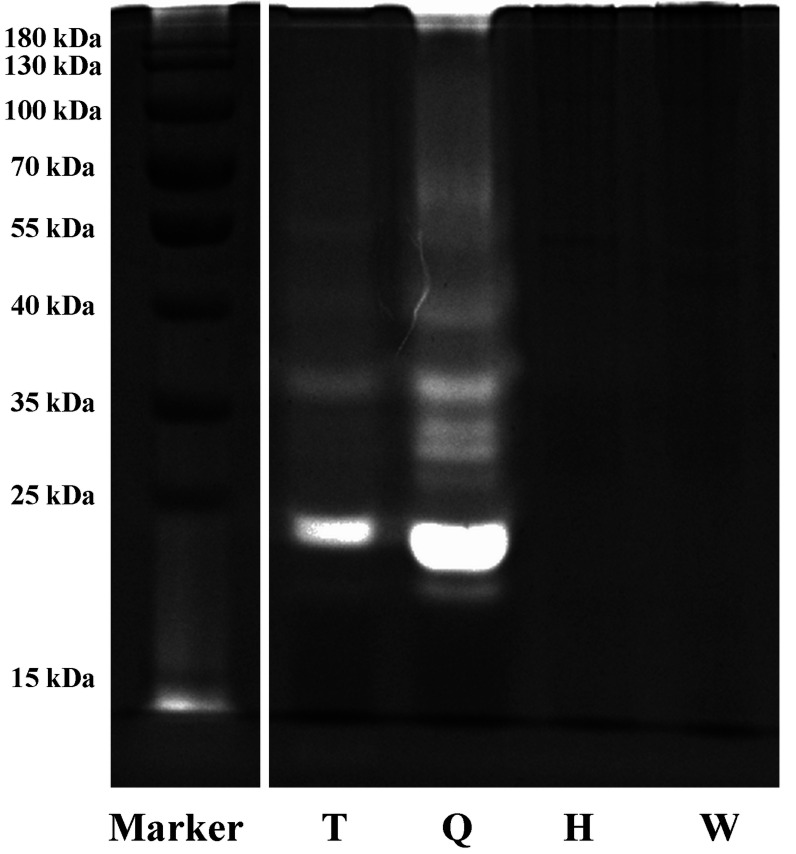
The fibrinogen zymogram of different PA portions (T: anterior portion of the PA, Q: anterior-midportion of the PA H: mid-posterior portion of the PA, W: posterior portion of the PA). Fibrinogen zymography was carried out using a 12% polyacrylamide gel that had been prepared in the presence of fibrinogen (0.5 mg/mL) and thrombin (0.012 BP/mL). We loaded 2 μL samples (5 mg/mL) that were diluted with sample loading buffer without reducing agent into a fibrin gel.

In this study, fibrinogen zymography was applied to reveal the fibrinolytic proteins pheretima. Fibrinogen zymography, a branch of zymography to study hydrolytic enzymes based on substrate degradation, was used to determinate plasmin activity in gel. In the fibrinogen zymography experiment, LKs were separated by their molecular weights and detected by their abilities to degrade the substrates. This technique lends itself to the analysis of any proteins with plasmin activity. The proteins possessing fibrinolytic activity could be detected as sharp and bright bands on the fibrinogen zymogram, which was visible to the naked eye. The result showed in Figure 3 revealed that clear bands appeared in the T and Q sample lanes, suggesting that LKs are distributed regionally in the anterior and anterior-mid, especially rich in the anterior-mid portion of PA, with the molecular weight from 15 kDa to 55 kDa. However, there was no significant fibrinogenolytic activity observed in the H and W lanes.

#### 2.1.3. Sample Preparation

Phenol-based extraction (PBE) and the ammonium acetate/methanol precipitation method was initially investigated for the preparation of total protein in PA [8,29]. The proteins extracted by PBE protocol did not possess fibrinolytic activity, and their digestion products indicated that Glu-C was specific on the carboxy terminal of Glu and Asp residues. On the reverse, the proteins extracted by ammonium bicarbonate solution showed fibrinolytic activity and there was hydrolysis of Ser-Cys, Ser-Ala, Ser-Leu, Leu-Leu, and Thr-Leu peptide bonds in samples. Furthermore, non-specific cleavages decreased when the earthworm protein digested with Glu-C and the sulfhydryl reduction reaction temperature increased from 60 °C to 95 °C. Ultimately, the extraction of PA protein was optimized by using a method of combining the ammonium bicarbonate and phenol-based extraction (AB-PBE) methods, and the sulfhydryl reduction reaction was at 95 °C. The peptides did not conform to the target peptide selection regulations that peptides of 6–25 amino acids were preferred, when only Glu-C was used as the hydrolase. Hence, we developed an efficient protocol, a two-step enzymatic hydrolysis method (trypsin/Glu-C), for proteins digestion from the PA.

#### 2.1.4. Discovery of Potential Biomarkers in PA

**Figure 4:**
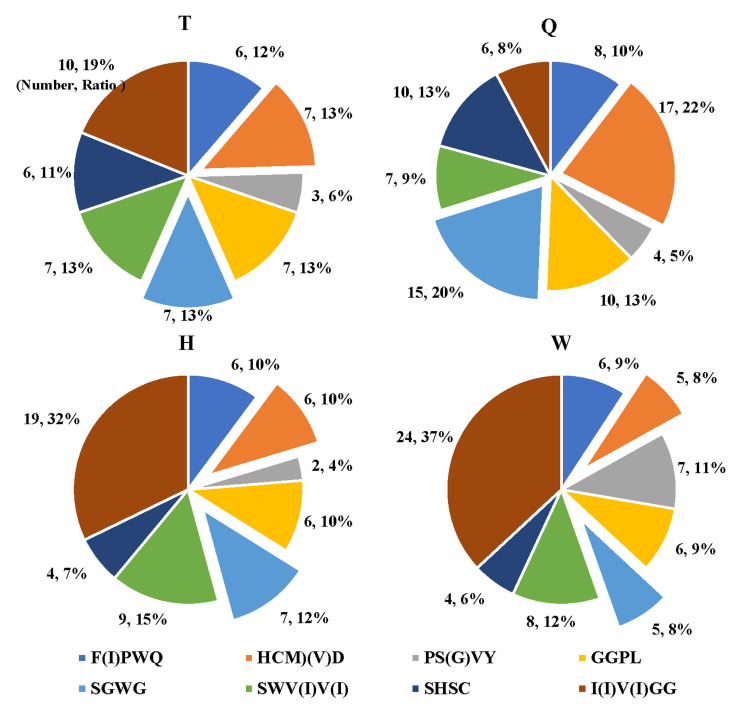
The number and ratio of target amino acid residues in T, Q, H, and W samples (The meanings of T, Q, H, and W are the same as those in Figure 3). The raw data were performed using Thermo Scientific Proteome Discoverer™ 2.2 software. MS/MS spectra were searched in the theoretical pheretima protein database obtained by transcriptomics (including 240,102 proteins). We detected 53, 77, 59, and 65 target peptides with shotgun proteomics from T, Q, H, W samples of PA, respectively.

Considering the sequence differences between the PA fibrinolytic protein and the known LKs due to species evolution, discovery proteomics was used to confirm theoretical homologous peptides in different species. The results showed more peptides containing SGWG amino acid residues, compared to peptides contained FPWQ and PS(G)VY. Moreover, peptides containing amino acid residues -HCMQ were not detected in the samples, but peptides containing HCMD amino acid residues were in high abundance. Candidate peptides were optimized according to the search results of LC-MS/MS experiments against the protein database of PA, not directly employed peptides digested in silico. The target peptides, containing I(V)V(I)GG, F(I)PWQ, SHF(S)C, HCMD(VD), SGWG, GGPL, SWV(I)V(I) and PS(G)VY amino acid residues, were selected according to sequence alignment results combined with shotgun proteomics results. In total, we detected 53, 77, 59, and 65 target peptides with shotgun proteomics from T, Q, H, W portions, respectively. Figure 4 shows the proportion of target amino acids in each sample. The number of peptides containing HCMD(VD) and SGWG was the most in portion Q. Peptides in the T and Q portions were selected as the target peptides, since the two portions possessed major fibrinolytic activity in the zymography experiment (Figure 3). It should be noted that there were common peptides in the T and Q portions, and ultimately 86 peptides were selected by excluding redundancy, among which 73 peptides containing 6 to 25 amino acids were kept as potential marker peptides in PA associated to fibrinolytic activity (see Table S1 in Supplementary Materials).

### 2.2. PRM Data Processing for Peptide Markers Quantitation

**Figure 5:**
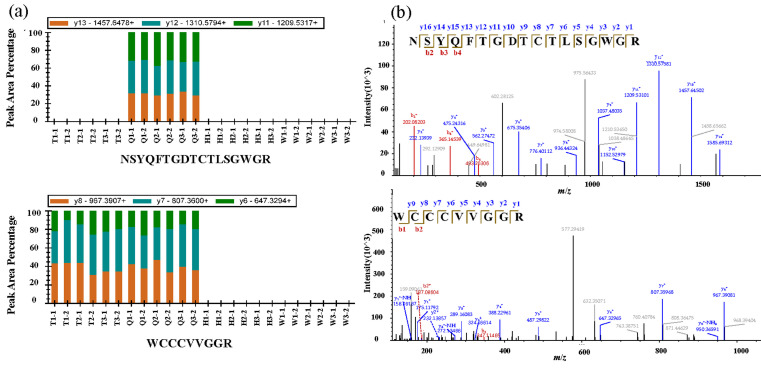
(**a**) The normalized peak area of marker peptides in T, Q, H, and W samples with three replicates. (**b**) MS/MS spectra for the target marker peptides taking peptides NSYQFTGDTCTLSGWGR and WCCCVVGGR as examples (the meanings of T, Q, H, and W are the same with those in Figure 3). The raw data was processed using Skyline (Version 3.6.0) with the software settings according to the online tutorials (https://skyline.ms/wiki/home/software/Skyline/page.view?name=tutorial_prm, accessed on 5 March 2021). The peptide search results of DDA data from Proteome Discoverer analysis were used for spectral library building in skyline.

To validate peptide markers, PRM assays were developed to both qualify and quantify specific candidate peptides from LKs. Featured as a method with high sensitivity, specificity, and reproducibility, PRM is an increasingly popular alternative technique in targeted proteomics [30,31,32]. In PRM, the peptide fragments were analyzed in an Orbitrap mass detector and yielded highly reliable peptide identity. For each precursor ion the *m*/*z* value selected corresponded to the major charge state (z = 1, 2). The transitions in each case were chosen using the three most abundant fragments. The peak area was normalized to “total” to visualize the presence or absence of different peptides. By tracing these transitions for each peptide marker presented in Table S1, it was possible to unequivocally distinguish T, Q, H, and W portions. Combined with the results from fibrinogen zymography that fibrinolytic activity was distributed in the T and Q portions, peptides present in the T and Q portions but not in the H and W samples could be considered the markers representing fibrinolytic activity. Eleven peptides were characterized related to fibrinolytic activity, among which 10 peptides (including NSYQFTGDTCTLSGWGR, IPWQLSQQR, ASPGEFPWQLSMTR, ALCAAHCVD, LWVVTAAHCMDGE, SHFCGGSIIND, GGSHSCGATLLSGTR, LPANNNNQYVGLICQISGWGR, GNSGACNGDSGGPLNCPDGVTR, and GGSHSCGASLLHATAALSAAHCVD) were detected in the Q portion, and 1 (WCCCVVGGR) was found in both T and Q portions. Detailed information about the 11 peptides was described in the Supplementary Material (Figure S1). The signal distribution of target ions and the corresponding fragment spectra in T, Q, H, and W portions is illustrated in Figure 5, using peptides NSYQFTGDTCTLSGWGR and WCCCVVGGR as examples.

The 11 peptide markers matched to 13 proteins, 12 of which were relevant to fibrinolytic activity according to the descriptions in non-redundant protein sequences (NR) database of NCBI (Table 1). By blasting the sequences of the 12 proteins with those of known LKs reported in uniprotKB, 8 LKs were retrieved, and the confidence level was from 51.5% to 76.1% (Table S2 in Supplementary Materials). Therefore, the fibrinogen zymography combined with PRM experiments showed that only the active portion of earthworm contained the corresponding marker peptides, suggesting that these peptides could characterize the fibrinolytic activity or the therapeutic effect of earthworm. It is believed that these marker peptides associated with fibrinolytic efficacy would have better potential in quality control of pheretima to ensure its effectiveness and stability in the clinical applications.

## 3. Discussion

Ideal sample preparations should evenly solubilize all target proteins and eliminate all other biological compounds that could potentially interfere with the experiment process. There is no universal way for perfect sample prep; rather, each sample type needs a sample preparation protocol optimized for itself. In the study, PA protein extracted with ammonium bicarbonate solution was with non-specific cleavage sites. For example, the Ser-Cys, Ser-Ala, Ser-Leu, Leu-Leu, and Thr-Leu bonds were hydrolyzed in the protein digestion process. Fortunately, it was found that there was a decrease in non-specific cleavage when the sulfhydryl reduction reaction temperature increased from 60 °C to 95 °C. Therefore, we speculated that most of these cleavages might be attributed to the action of plasmin activities associated with LKs, which were heat-resistant and cannot be inactivated when heated at 60 °C for 1 h [33,34]. Ultimately, the extraction of PA protein was optimized by using a method combining the ammonium bicarbonate and phenol-based extraction (AB-PBE) methods, and the sulfhydryl reduction reaction was at 95 °C.

Since the amino acid sequences of LKs possessed a high abundance of proline and a low abundance of arginine and lysine, trypsin was not a good choice for LKs digestion. Glu-C was selected as the candidate proteases by in silico digestion. But the experimental data indicated that some candidate peptides possessed more than 25 amino acids and contained trypsin cleavage site, which disagreed with those obtained from the in silico analysis. Ultimately, the combination of trypsin and Glu-C digestion was determined to be the most suitable for this experiment.

A PRM workflow was developed and optimized to improve the identification of marker peptides associated with fibrinolytic activity, based upon the knowledge that LKs, isozymes isolated from different species of earthworm, have similar amino acid sequences. The conserved amino-acid sequences screened from PA were slightly different from those from LKs, which suggested that the conserved amino-acid residue differ between experimental and theoretical analysis as substitution of the amino acids with similar structure and functional ones at the same site. This condition should be appreciated, or some potential marker peptides would be ignored.

Thanks to the PRM method having high sensitivity and specificity for the target ions, the peptides even with very low abundance could be unambiguously identified in the samples. In addition, a few peptides were detected in individual H, W portions, but they were very low compared with those in Q samples, indicating no fibrinolytic activities. In this paper, the main focus was on the presence or absence of marker peptides related to fibrinolytic activity in different parts of PAs, and the peptides with significantly different content will be studied in the follow-up research.

Pheretima collected from TCM markets showed a significant difference in fibrinolytic activities. Moreover, different slices from the same batch samples sometimes showed a very significant difference in daily detection. Zhao found that fibrinolytic activity was mainly detected around the clitellum of *Eisenia fetida* on fibrin plates [10]. In this study, fibrinogen zymography was applied to reveal the fibrinolytic activity and the molecular weight of fibrinolytic proteins of pheretima. The result showed that fibrinogenolytic activity was observed in T and Q samples but not in the H and W samples. This discovery suggested that half of the pheretima in the market had no fibrinolytic activity, which would provide a reference for the intelligent use of pheretima in the treatment of thrombosis-related diseases.

Pheretima has been recorded as a crude drug prescribed for anticoagulation, antipyretic and diuretic purposes for thousands of years. Unfortunately, hitherto the quality standards remained at a low level for poor specificity of these candidates, and few methods were available for detection due to their high polarity. Screening peptides as quality markers with label-free quantification proteomics has become an effective way to improve the quality control for pheretima. For example, species-specific marker peptides for pheretima were reported in a previous study [7,8]. Unfortunately, these strategies based on label-free proteomics were considered to be complicated and time-consuming for the difficulty in collection of multiple representative samples and the professional statistical analysis. As well, it is impossible to apply this method in the screening of active peptides. In the present study, a new proteomics strategy was proposed by combining prediction of the potential active peptides by bioinformatics analysis with validation of them by LC-MS/MS and activity assay experiments. Compared with the traditional method, this strategy would work more promptly and the efficacy-based quality assessment would be a powerful analytical method to ensure the effectiveness and stability in clinical applications. However, this study was based on fresh earthworm. Whether the screened marker peptides associated with fibrinolytic efficacy can be used to detect the biological activity of medicinal materials needs further study.

## 4. Materials and Methods

### 4.1. Chemicals and Materials

Pierce Quantitative Fluorescent Peptide Assay, Pierce BCA protein assay kit, Page Ruler prestained protein ladder, Pierce C18 Tips, acetonitrile (LC/MS grade) and formic acid (LC/MS grade) were purchased from Thermo Fisher Scientific (Waltham, MA, USA). Protease Inhibitor Cocktail and endoproteinase Glu-C (Sequencing grade) were purchased from Roche (Mannheim, Germany). Phenol solution (pH 7.9 ± 0.2) was purchased from Sangon Biotech (Shanghai, China) Co., Ltd., Genomic. Ultrapure water (18.2 MΩ.cm) was prepared in-house by a Milli-Q Advantage A10 water purification system (Millipore, Bedford, MA, USA). Sequencing-grade modified trypsin and molecular weight cutoff membranes of 10 kDa were purchased from Promega (Promega Corporation, Madison, WI, USA).

*P. aspergillum* was collected from markets and identified using conventional polymerase chain reaction (PCR) to amplify the cytochrome C oxidase subunit I (COI) gene [35].

### 4.2. Protein Extraction

PA was cut into four segments as benchmark samples, including anterior (T), anterior-mid (Q), mid-posterior (H), and posterior (W) portions. The samples were pelleted at 3000*× g* for 10 min before protein extraction. About 10 mg of the pretreated samples were extracted by sonication in 200 microliter ammonium bicarbonate buffer (50 mM) at 37 °C for 40 min. The supernatant was collected by centrifugation at 14,000*× g* at 4 °C for 10 min, and then filtered with molecular weight cutoff membranes of 10 kDa to obtain the retentate, which was used directly for fibrinogen zymography tests.

Protein for proteomic tests was prepared separately. Retentate was resuspended in 200 μL lysis buffer containing 1% SDS, Tris-HCl (pH 8.6, 100 mM), EDTA (10 mM), sodium borate (5 mM), DTT (5 mM), 1× cOmplete protease inhibitors (Roche Complete mini EDTA free, Mannheim, Germany) buffer. The solution was mixed with eight volumes of ice-cold ammonium acetate/methanol (0.1 M). Precipitates were pelleted by centrifugation at 14,000*× g* at 4 °C for 10 min, subsequently washed twice with 90% ice-cold acetone and air-dried for 5 min. Then the precipitate and purify protein was dissolved in rehydration buffer (8 M urea containing 1% SDS) for the further study and the protein concentration was determined by BCA assay.

### 4.3. Fibrinogen Zymography

The specific proteolytic activity of samples was confirmed with fibrinogen zymography. Zymography was based on a sodium dodecyl sulfate polyacrylamide gel impregnated with protein substrate that was later degraded by the proteases during incubation. The fibrin zymography protocol was similar to the previous report with some modifications [36,37]. Resolving gel solution (12%) contained fibrinogen and Thrombin in final concentrations of 0.5 mg/mL and 0.012 BP/mL, respectively. Samples of T, Q, H, W portions were electrophoresed into the fibrin gel and subsequently washed with 2.5% Triton X-100 solution for 30 min under gentle agitation. The gel was then incubated in a bath containing PBS at 37 °C for 30 min. After incubation, it was stained in Coomassie blue R250 staining solution overnight and distained in solution containing methanol–acetic acid–water (1:1:8, *v*:*v*:*v*).

### 4.4. Bioinformatics

LKs isolated from different species of PAs are isozymes. To further understand the protein sequence similarity in LKs, a total of 36 LKs sequences were downloaded from the UniProtKB protein database (http://www.uniprot.org/, accessed on 10 May 2020) and NCBI website (https://www.ncbi.nlm.nih.gov/, accessed on 10 May 2020) and blasted using the MEGA7 software to spot identical sequences. The online program Expasy Peptide Cutter (https://web.expasy.org/peptide_cutter/, accessed on 3 June 2020) was used as a bioinformatics tool for in silico digestion. The subsequence peptides were integrated into a small database. Next, the coverage of these peptides in the nano-LC-MS/MS experiments was examined by searching against proteins from a local theoretical database containing 240,102 sequence entries of *P. aspergillum* and *P. vulgaris*, constructed via RNA-Seq technology. The database was downloaded from the U.S. National Center for Biotechnology Information (NCBI) website (http://www.ncbi.nlm.nih.gov/sra/, TaxID: 320991, TaxID: 437222, accessed on 3 December 2018).

### 4.5. Digestion

About 200 μg PA protein was dissolved in 200 μL of ammonium bicarbonate buffer (50 mM) with DTT (10 mM) at 95 °C for 20 min in a shaker. The alkylation was performed with iodoacetamide (20 mM) at room temperature in darkness for 30 min, followed by added DTT (10 mM) to consume the excess iodoacetamide. The mixtures were concentrated using Amicon Ultra 10 kDa centrifugal filters (Millipore, Billerica, MA, USA) at 14,000*× g* for 20 min at 4 °C and washed with 400 μL of ammonium bicarbonate buffer twice. The protein digestion was conducted in diverse conditions: (i) For endoproteinase Glu-C (Glu-C) digestion, proteins were digested using sequencing grade modified Glu-C in phosphate buffered solution (pH 7.8, 50 mM) at an enzyme-to-protein ratio of 1:50 (*w*/*w*) at 45 °C for 4 h. (ii) For trypsin/Glu-C digestion, trypsin was added first at an enzyme-to-protein ratio of 1:50 (*w*/*w*) reacting in ammonium bicarbonate (50 mM) at 37 °C for 18 h. The whole digest was lyophilized under vacuum, and followed by Glu-C digestion at a ratio of 1:50 (*w*/*w*) in PBS buffer (pH 7.8, 50 mM) at 45 °C for 4 h. All the digestions described above were terminated by adding 10% formic acid to pH 2. The protein lysate was desalted with C18 tip and lyophilized under vacuum. The dry powders were dissolved in 30 μL 0.1% formic acid for LC-MS analysis.

### 4.6. Shotgun LC-MS/MS Analysis

The samples from the same individual were complex and contained many proteins without fibrinolytic activity. It is crucial to detect unique peptides in complicated backgrounds with an MS instrument. Digested samples were analyzed by UltiMate 3000 RSLC nano system (Thermo Fisher Scientific, Sunnyvale, CA, USA) coupled to an Orbitrap Fusion Lumos mass spectrometer (Thermo Fisher Scientific, San Jose, CA, USA). The separation of the peptides was carried out on an Acclaim PepMap100 C18 trap Column (5 μm, 100 Å, 100 μm × 1 cm) (Thermo Fisher Scientific, Waltham, MA, USA) coupled to an RP analytical column Acclaim PepMap RSLC 75 μm × 150 mm, C18, 2 μm, 100 Å (Thermo Fisher Scientific, Waltham, MA, USA), using mobile phase (A) of 0.1% formic acid in water and mobile phase (B) of 80% ACN with 0.1% formic acid. A linear gradient of 90 min from 4 to 35% B, at a flow rate of 300 nL/min was used. For ionization, a spray voltage of 2 kV and a capillary temperature of 275 °C were used, and the tandem mass spectra were acquired using data-dependent acquisition (DDA). Mass spectrometry analysis of raw data was conducted with Proteome Discoverer 2.2 software (Thermo Fisher Scientific, Waltham, MA, USA). The general settings were as follows: mass analyzer was Fourier transform mass spectrometry, MS order was MS^2^, activation type was HCD using a normalized collision energy of 30%, polarity mode was positive, enzymes were trypsin and Glu-C, precursor mass tolerance was 10 ppm, fragment mass tolerance was set to 0.02 Da, dynamic modifications (peptide terminus) were oxidation/+15.995 Da (M), static modifications were carbamidomethyl/+57.021 Da (C). The database was the local theoretical protein database (including 240,102 proteins).

### 4.7. PRM Mass Spectrometry

PRM mass spectrometry was carried out by Orbitrap Fusion Lumos mass spectrometer in PRM mode with the parameters similar with shotgun LC-MS/MS analysis. The data were collected in three replicates. The PRM data were processed using Skyline (Version 3.6.0) with the software settings according to the online tutorials (https://skyline.ms/wiki/home/software/Skyline/page.view?name=tutorial_prm, accessed on 5 March 2021). The peptide search results of DDA data from Proteome Discoverer analysis were used to build a spectral library for skyline.

## 5. Conclusions

An efficient strategy was proposed in this work by integration of bioinformatics and PRM to rapidly screen marker peptides associated with fibrinolytic activity of pheretima, a typical animal-derived traditional medicine. Consequently, 11 discriminable peptides were screened out for detection of fibrinolytic activity in different portions of PA, which could be regarded as marker peptides for the quality evaluation of pheretima. The obtained results also indicated that LKs accumulated in the head and the anterior portion of the PA, especially in anterior-midportion, and there was minimal fibrinolytic activity found in the mid-posterior and posterior portion. This discovery suggested that half of the pheretima in the market had no fibrinolytic activity, which would provide a reference for the intelligent use of pheretima in the treatment of thrombosis-related diseases. We anticipate the marker peptides would help improve the quality standards related to fibrinolytic activity of pheretima, and the workflow established in this work will also benefit the study of other animal-derived traditional medicines.

## Figures and Tables

**Table 1:** Peptide markers associated with thrombolysis efficacy of *P. aspergillum*.

Peptide Sequence	*m*/*z*	Charge	Sequence Length	Distribution	Proteins Accession	Proteins Description
NSYQFTGDTCTLSGWGR	975.4260	2	17	Q	DN78691_c1_g2_i2::g.671430	fibrinolytic enzyme
					DN109923_c9_g2_i1::g.21772	fibrinolytic enzyme
IPWQLSQQR	1155.6269	1	9	Q	DN94857_c2_g1_i9::g.607734	lumbrokinase
					DN94857_c2_g1_i6::g.607728	lumbrokinase
ASPGEFPWQLSMTR	803.8878	2	14	Q	DN84311_c4_g1_i3::g.903727	lumbrokinase-7T2 precursor
					DN84311_c4_g1_i4::g.903729	lumbrokinase-Da2 precursor
ALCAAHCVD	1016.4288	1	9	Q	DN84311_c4_g1_i3::g.903727	lumbrokinase-7T2 precursor
					DN84311_c4_g1_i6::g.903732	lumbrokinase-Da2 precursor
					DN84311_c4_g1_i4::g.903729	lumbrokinase-Da2 precursor
					DN84311_c4_g1_i7::g.903735	fibrinolytic enzyme component A
					DN83697_c1_g5_i1::g.656593	lumbrokinase-7T2 precursor
LWVVTAAHCMDGE	744.8341	2	13	Q	DN102309_c1_g2_i5::g.177583	lumbrokinase
					DN102309_c1_g2_i1::g.177575	lumbrokinase
SHFCGGSIIND	1206.5208	1	11	Q	DN102309_c1_g2_i5::g.177583	lumbrokinase
					DN102309_c1_g2_i1::g.177575	lumbrokinase
GGSHSCGATLLSGTR	730.8492	2	15	Q	DN83697_c1_g5_i1::g.656593	lumbrokinase-7T2 precursor
LPANNNNQYVGLICQISGWGR	792.0622	2	21	Q	DN83697_c1_g1_i5::g.656587	lumbrokinase-7T1 precursor
GNSGACNGDSGGPLNCPDGVTR	721.3044	2	22	Q	DN84311_c4_g1_i7::g.903735	fibrinolytic enzyme component A
GGSHSCGASLLHATAALSAAHCVD	784.0269	2	23	Q	DN109923_c9_g2_i1::g.21772	fibrinolytic enzyme
WCCCVVGGR	577.2386	2	9	T, Q	DN87155_c0_g4_i1::g.790308	hypothetical protein

## Data Availability

Data is contained within the article or Supplementary Material.

## Supplementary Materials

The following are available online at https://www.mdpi.com/article/10.3390/molecules27092651/s1, Figure S1: The signal distribution of 11 quality markers and the corresponding mass spectra in T, Q, H, and W samples. Table S1: Potential marker peptides associated with fibrinolytic activity of *P. aspergillum*. Table S2: Blast results of matched proteins in UniProtKB.

## Sample Availability

Samples of the compounds are available from the authors.
