# Supplementary material for: A Strategy for Rapid Discovery of Marker Peptides Associated with Fibrinolytic Efficacy of Pheretima aspergillum Based on Bioinformatics Combined with Parallel Reaction Monitoring
Source: Molecules. 2022 Apr 20;27(9):2651. doi: 10.3390/molecules27092651 (PMC9100157; doi:10.3390/molecules27092651)
Supplement: Supplementary file 1 [file molecules-27-02651-s001.zip › molecules-1677059-supplementary.pdf]

Supplementary Materials to the manuscript

A Strategy for Rapid Discovery of Marker Peptides Associated with Fibrinolytic Efficacy of *Pheretima aspergillum* Based on Bioinformatics Combined with Parallel Reaction Monitoring

Ting-Ting Feng [1,2,3], Jing-Xian Zhang [1], Yong-Peng Zhang [3], Jian Sun [1], Hong Yu [1], Xiang Tao [1], Xiu-Hong Mao [1], Qing Hu [1,*] and Shen Ji [1,2,*]

[1] NMPA Key Laboratory for Quality Control of Traditional Chinese Medicine, Shanghai Institute for Food and Drug Control, 1500 Zhangheng Road, Shanghai 201203, China

[2] China State Institute of Pharmaceutical Industry, 285 Gebaini Road, Shanghai 201203, China

[3] Institute of Traditional Chinese Medicine, Hebei North University, 11 Diamond South Road, Zhangjiakou 075000, China

* Correspondence: huqingyjs@163.com (Q.H.); jishen2013@163.com (S.J.)

Supplementary Materials figure

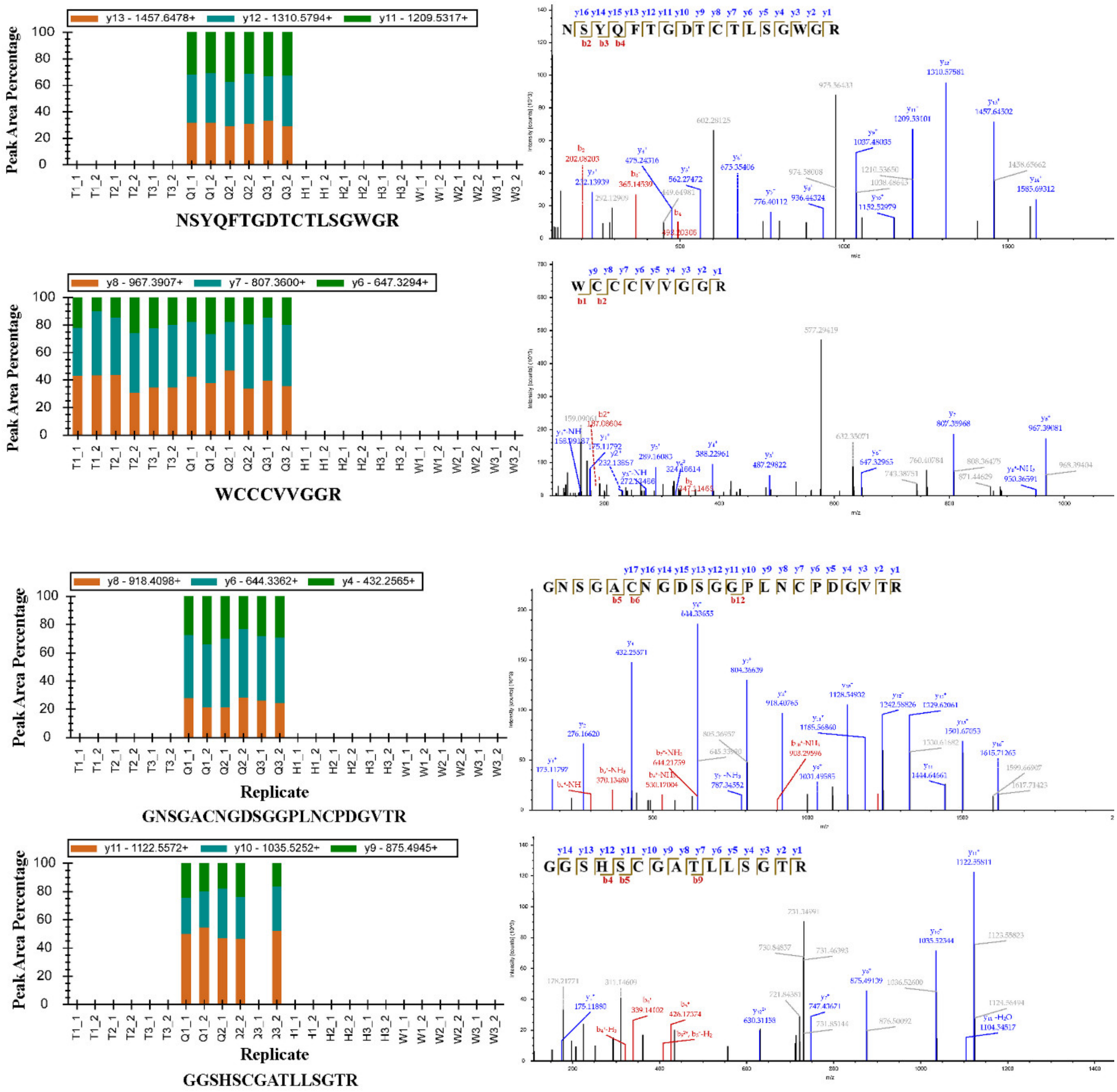

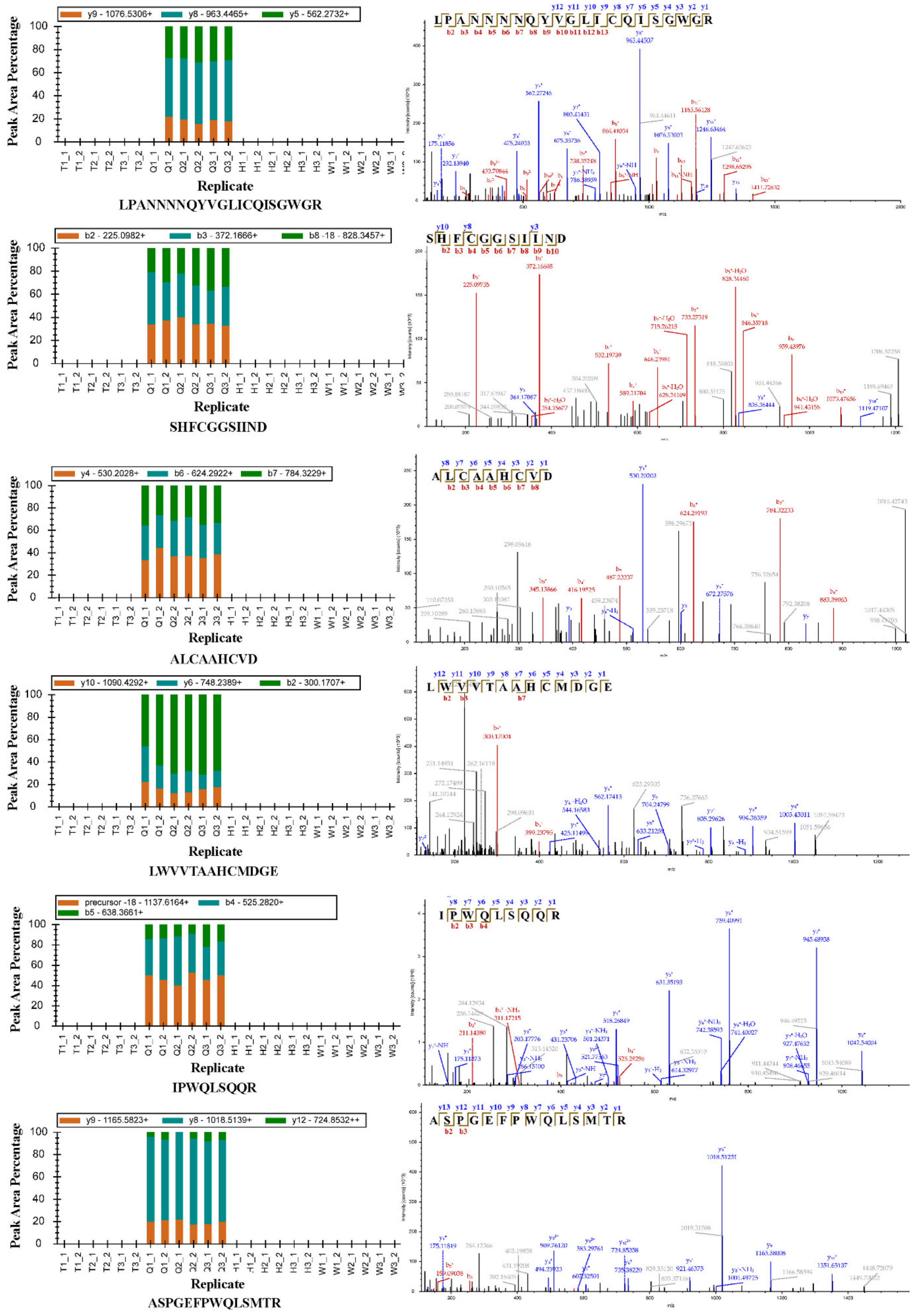
y9 - 1076.5306+
y8 - 963.4465+
y5 - 562.2732+
Peak Area Percentage
Replicate
LPANNNNQYVGLICQISGWGR
b2 - 225.0982+
b3 - 372.1666+
b8 -18 - 828.3457+
SHFCGGSIIND
y4 - 530.2028+
b6 - 624.2922+
b7 - 784.3229+
ALCAAHCVD
y10 - 1090.4292+
y6 - 748.2389+
b2 - 300.1707+
LWVVTAAHCMDGE
precursor -18 - 1137.6164+
b4 - 525.2820+
b5 - 638.3661+
IPWQLSQQR
y9 - 1165.5823+
y8 - 1018.5139+
y12 - 724.8532++
ASPGEFPWQLSMTR

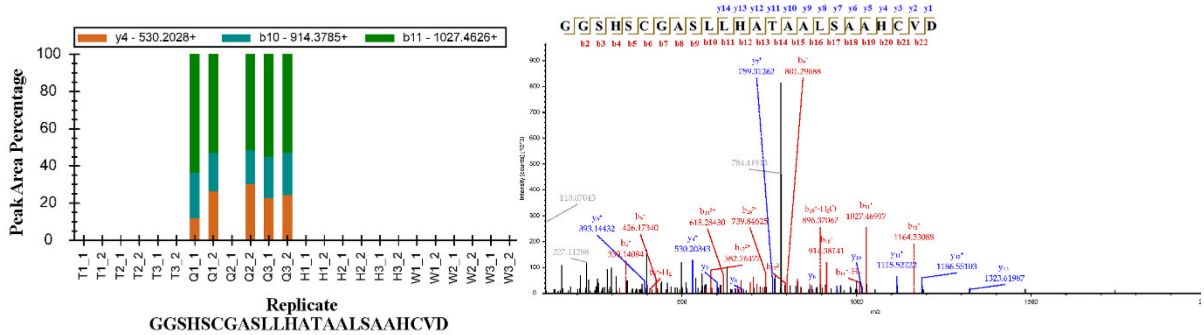

Figure S1. The signal distribution of 11 quality markers and the corresponding mass spectra in T, Q, H, and W samples. The raw data was processed using Skyline (Version 3.6.0) with the software settings according to the online tutorials (https://skyline.ms/wiki/home/software/Skyline/page.view?name=tutorial_prm). The peptide search results of DDA data from Proteome Discoverer analysis were used for spectral library building in skyline.

Supplementary Materials tables

Table S1. Potential marker peptides associated with fibrinolytic activity of P. aspergillum.

Compound	m/z	t start (min)	t stop (min)
AGYNMLFVGIGGPLIPCER	688.6841	62	90
ALCAAHCVD	1016.4280	15	39
ALCAAHCVDGALTSSLAVIAGLHNR	645.0802	50	80
ALCAAHCVDGALVSSLAVIAGLHNR	859.1113	53	83
ARPYEFPWQVSVR	817.9253	41	71
ASPGEFPWQLSMTR	803.8867	49	79
AVTAAHCSGSATGVYSLLGGTSDR	1169.5575	33	63
CGGCTVIGGCR	598.7515	15	41
DGTGSCDPSYPSVYTR	881.3718	30	60
DGTWYQAGLTSWGVVNCK	1021.4744	54	84
DSCQGDSGGPLSVK	703.8131	17	47
DSCQGDSGGPLTVK	710.8217	16	46
FALLPPDNVEQFVGFTCVLSGWGR	1355.1771	63	90
FGGPLICPDFTE	676.8199	54	84
FPWQLSLQR	587.8216	55	85
FPWQVSVR	1018.5458	39	69
FVVAGVTSWGISGGGACLPE	982.4819	61	90
FVVAGVTSWGISGGGACLPEYPSVY	1287.1221	63	90
GACDGDFGGPLICPDFTE	964.3948	57	87
GANCDISGWGK	582.7578	21	51
GEFPWQLSMTR	676.3255	48	78
GGNCDISGWGK	575.7501	20	50
GGPLIDPSK	883.4875	15	45
GGSHSCGASLLGSTHALCAAHCVD	809.0195	26	56
GGSHSCGASLLHATAALSAAHCVD	784.0259	30	60
GGSHSCGASLLR	601.2902	15	38
GGSHSCGASLLRPGSAL	813.9033	24	54
GGSHSCGATLLSGTR	730.8484	15	44
GGSHSCGATLLSSTR	745.8534	15	42
GIHIPGSPFQFTVGPIVGGGPEK	764.4097	52	82

GNSGACNGDSGGPLNCPDGVTR	721.3037	20	50
GSVIAGVGSWVVR	643.8639	44	74
IAILPPDNSYQFTGDTCTLSGWGR	890.4271	55	85
IGIIGGTGLDNPDLLQER	941.0075	48	78
IPWQLSQQR	1155.6233	29	59
IVGGSAVSISDYPHQLSMR	1009.0125	35	65
IVGGSDAGIGSYK	612.3160	20	50
LATVSDGPFEGGNCDISGWGK	1083.9917	43	73
LFDFLHCVDHCVSK	592.9436	40	70
LLSGITSWGVANCSPAWPSVYTR	841.4205	57	87
LPANNNNQYVGLICQISGWGR	792.0611	58	88
LSSGTWSHSCGATLLSAR	630.9751	30	60
LWVVTAAHCMD	651.8015	38	68
LWVVTAAHCMDGE	744.8336	41	71
LWVVTAAHCMDGESPAQVSIVAGE	843.0689	52	82
NSYQFTGDTCTLSGWGR	975.4261	41	71
PGSALSAAHCVD	592.7712	15	45
PGSALSAAHCVDGAPPADVR	649.9821	22	52
PYEFPWQVSVR	1407.7057	49	79
QFVGFTCVLSGWGR	807.3969	57	87
QVVDTVVGGNPDR	678.3480	19	49
QWIVTAAHCMDGEVPSQVSLVVGE	1306.6289	55	85
SGGPLDSSGFE	1052.4517	34	64
SGGPLNCPDGVTR	665.3135	17	47
SHFCGGSIIND	1206.5187	23	53
SHFCGGSIINE	610.7713	24	54
SHSCGASLLR	544.2686	15	38
SQCSGWGSLSSGGVCCPAVLR	742.3344	42	72
SQCSGWGTLQSGGACCPD	964.3707	34	64
SSDSHFCGGSIINE	755.3167	27	57
SSWGVGASVLTK	596.3212	33	63
STDSHFCGGSIIND	755.3162	26	56
TCTLSGWGR	1037.4817	23	53
TLSGWGR	776.4044	18	48
VAGVTSWGISGQLGR	744.4013	39	69
VGEIPWQLSQQR	720.8814	38	68
VTGSHSCGASLISNTR	823.8975	15	42
WCCCVVGGR	577.2381	21	51
YGLSASHCVDGATADILR	635.9749	33	63
FPGQQIIGGELK	643.8588	49	64
SWGIGASATTR	553.7827	32	47
SWGIGATASTR	553.7827	32	47
YHFHYRPHEPGVYLLNVK	757.0596	38	53

Table S2. Blast results of matched proteins in UniProtKB

Matched Proteins Accession	Lumbrokinase Name /Accession a	Identity (%)
TRINITY_DN78691_c1_g2_i2::g.671430	Fibrinolytic enzyme (Enchytraeus japonensis) /H1A7B6	60.5
TRINITY_DN109923_c9_g2_i1::g.21772	Lumbrokinase (Eisenia fetida) /H1A7B5	62.7
TRINITY_DN94857_c2_g1_i9::g.607734	Fibrinolytic enzyme (Enchytraeus japonensis) /H1A7B6	55.2
TRINITY_DN94857_c2_g1_i6::g.607728	Lumbrokinase (Eisenia fetida) /A8ILP1	51.5
TRINITY_DN84311_c4_g1_i3::g.903727	Lumbrokinase (Eisenia fetida) /A8ILP1	68.6
TRINITY_DN84311_c4_g1_i4::g.903729	Lumbrokinase (Eisenia fetida) /Q5MBA0	68.6
TRINITY_DN84311_c4_g1_i6::g.903732	Lumbrokinase-6 (Eisenia fetida) /Q6T373	65.4
TRINITY_DN84311_c4_g1_i7::g.903735	Lumbrokinase-6 (Eisenia fetida) /Q6T373	67.9
TRINITY_DN83697_c1_g5_i1::g.656593	Lumbrokinase (Eisenia fetida) /A8ILP4	68.2
TRINITY_DN102309_c1_g2_i5::g.177583	Lumbrokinase (Perionyx excavatus) /A0A068B4B1	76.1
TRINITY_DN102309_c1_g2_i1::g.177575	Lumbrokinase (Eisenia fetida) /Q6DKQ2	76.2
TRINITY_DN83697_c1_g1_i5::g.656587	Lumbrokinase (Eisenia fetida) /A8ILP1	68.9

a: Lumbrokinase with the highest matched identity degree in UniProtKB
